# Expenditures on Strengthening Large Scale Breastfeeding Counseling Programs in Bangladesh, Ethiopia, and Vietnam

**DOI:** 10.1111/mcn.70031

**Published:** 2025-04-04

**Authors:** Tina G. Sanghvi, Rick Homan, Tuan Nguyen, Zeba Mahmud, Marina Nersesyan, Patricia Preware, Edward A. Frongillo, Roger Mathisen

**Affiliations:** ^1^ Alive & Thrive initiative, FHI 360, Family Health International, Washington DC and Durham North Carolina USA; ^2^ FHI 360, Health Services Research, Global Health and Population Durham North Carolina USA; ^3^ FHI 360, Alive & Thrive Hanoi Vietnam; ^4^ FHI 360, Alive & Thrive Dhaka Bangladesh; ^5^ Department of Health Promotion, Education, and Behavior University of South Carolina Columbia South Carolina USA

**Keywords:** Bangladesh, breastfeeding counseling, budget expenditures, Ethiopia, interpersonal communication, large scale programs, Vietnam

## Abstract

Timely support given to breastfeeding mothers can result in life‐saving benefits for both mothers and infants. Progress in achieving results from existing efforts to improve breastfeeding practices can be accelerated with adequate investments in effective interventions. We aimed to document expenditures incurred by three diverse programs in Bangladesh, Ethiopia, and Vietnam that demonstrated improved breastfeeding outcomes. Based on expenditure records, we retrospectively calculated annual and per participant expenditures. The results represent the incremental financial needs of strengthening existing efforts in low‐ and middle‐income countries to inform budget planning. The programs reached between 400,000 to 1.2 million pregnant women, infants, and mothers annually at an average expenditure of USD 0.55 to 1.90 per woman and infant. The largest proportion of expenditures were incurred for training frontline workers and delivering interpersonal communication or counseling. These ranged from 73.4% of total expenditures in Bangladesh to 63.9% in Ethiopia and 55.1% in Vietnam. Management and administration expenditures ranged from 13.3% and 19.6% across countries; the range in expenditures for planning and strategy development was 2.5%–9.9%; for materials development and production was 1.1%–15.1%; and for monitoring was 1.7%–18.7%. The results show that existing cadres of facility and community workers can deliver effective breastfeeding counseling on a large scale with substantial economies of scale. Budgetary needs will differ by country due to delivery system strengths and weaknesses, pre‐existing coverage, and demand for counseling services. The study provides a basis for realistic budget estimates for strengthening breastfeeding counseling in large‐scale programs.

## Summary

- Expenditures on strengthening existing breastfeeding services ranged from USD 0.55 to USD 0.65 and USD 1.90 per woman and infant for Bangladesh, Ethiopia, and Vietnam respectively. Half to three‐quarters of total expenditures were incurred for training frontline workers and delivering counseling services.
- Vietnam reached 400,000 women and infants annually through facilities, Ethiopia reached 720,000 women and infants through facilities and community‐based approaches, and Bangladesh reached 1.2 million women and infants through community groups and home visits.
- If countries use existing delivery systems and reach a large scale, the per participant financial needs of strengthening existing breastfeeding services can be affordable.

## Graphical abstract

The costs of enabling mothers' environment for breastfeeding through large‐scale programs that successfully address critical factors at multiple levels are only a fraction of the losses due to deaths and illnesses from poor breastfeeding practices. In Bangladesh, Ethiopia and Vietnam, the programs reached between 400,000 to 1.2 million pregnant women, infants, and mothers annually at an average expenditure of USD 0.55–1.90 per woman and infant. These programs focused on strengthening the health system and family/community networks with the understanding that mothers' breastfeeding practices depend upon her motivation, interactions with her infant, her self‐confidence, perception of social norms, and family support.

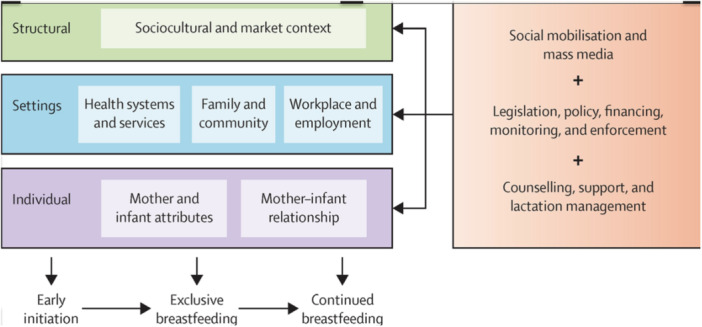

## 1 Introduction

Investments in protecting breastfeeding (BF) can deliver immediate and ongoing benefits for mothers, newborns, infants and young children, such as reduced risks of mortality and morbidity, improved cognition, and reduction in cancer and non‐communicable diseases in women (Chowdhury et al. 2015; Lamberti et al. 2013; Lee and Binns 2019; Sankar et al. 2015; Santiago et al. 2019). Timely support given to mothers at critical times starting in pregnancy can facilitate optimal practices (Kim et al. 2018; Louis‐Jacques and Stuebe 2020; McFadden et al. 2017; Sinha et al. 2015), but coverage of BF counseling is low (Değer et al. 2023; Hassounah et al. 2023; Lutter et al. 2011; Torlesse et al. 2021). The economic consequences of not investing in BF protection are enormous and deserve scrutiny especially in the development and health investment strategies of resource‐poor low‐ and middle‐income countries (LMIC) (Murthi and Shekar 2021; Pretorius et al. 2021). A critical gap in the literature is the absence of data on the expenditures to provide universal BF counseling as recommended by the World Health Organization (WHO 2018), based not on hypothetical scenarios but on field implementation of large‐scale BF counseling programs in LMICs. This gap may have contributed to delayed progress in obtaining adequate resources and achieving large scale coverage of BF counseling (Carroll et al. 2020). We address this issue in this paper. We analyzed interpersonal communication (IPC) interventions focused on counseling pregnant women (PW) and mothers that formed a part of multi‐component government‐led programs to improve BF practices in Bangladesh, Ethiopia, and Vietnam. We use the term IPC as a comprehensive term for all types of face‐to‐face interactions involving PW and mothers individually or in groups that may also involve other participants, and counseling as a specific type of IPC, usually individualized where trained providers offer problem‐solving and confidence‐building support to PW and BF mothers (WHO 2021).

Although cost and coverage projections of BF IPC have been attempted in the past, the actual expenditures incurred and coverage achieved of IPC interventions based on programs implemented at scale in LMICs have not been previously reported (Carroll et al. 2018). The results presented here strengthen the evidence for planning budgets and projecting coverage based on real program experience for the first time. Decision‐makers have access to studies on impacts and benefits of BF interventions, but they often question the potential high cost and workload requirements of delivering IPC including high quality BF counseling. With limited documentation of large‐scale BF counseling costs to guide them, they hesitate to make strategic budgetary allocations for achieving results. This paper describes interpersonal interventions scaled up through different types of health services, provides the expenditures and coverage achieved, and discusses the total and per participant expenditures across the three countries and factors influencing cost shares of program components.

In Bangladesh, Ethiopia, and Vietnam, BF IPC was one component of a larger social and behavior change program and was accompanied by mass media and public education through local media (Kim et al. 2016; Menon et al. 2016). IPC or face‐to‐face communication on BF was conducted by health workers in facility contacts and during home visits for antenatal care, postnatal care, and child health services. Group meetings with trained facilitators were held for mothers to receive additional motivation and peer support. Community mobilization events were implemented for engaging community opinion leaders and influential persons such as family members, peers, village/town elders, local doctors and health providers, and opinion leaders through individual and group meetings and video and theater shows. The strategies to improve breastfeeding practices were based on the socio‐ecological model of behavior change and aimed to not only deliver a certain number of messages through a certain number of contacts but to deliver results in terms of enabling mothers to follow recommended BF practices (Bronfenbrenner 1992; Fraser and Galinsky 2010). External rigorous evaluations showed that exclusive breastfeeding (EBF) increased in Bangladesh by 36.2 percentage points (pp), in Ethiopia by 9.4 pp, and in Vietnam by 27.9 pp (Kim et al. 2016; Menon et al. 2016).

In alignment with global recommendations (United Nations Children's Fund (UNICEF), World Health Organization 2021), the IPC interventions supported the following practices: initiate BF as soon as possible after birth within the first hour after delivery; obtain hands‐on support to facilitate newborns to initiate and mothers to establish BF and manage common difficulties; learn how to express breast milk as a means of maintaining BF in the event of temporary separation; be responsive to infants' cues for feeding, closeness and comfort; and not to give any food or fluids other than breast milk unless medically indicated. The modes of service delivery were adapted to country contexts in consultation with stakeholders and government authorities and aimed at high and frequent contacts with PW and mothers. The programs differed in how the content was conveyed, the number and types of contacts, and the frequency of exposures to IPC (Kim et al. 2020). The content of IPC addressed behavioral determinants (knowledge of how to practice each behavior, belief in benefits) and psychosocial factors (self‐efficacy, perception of social norms, and family support) contextualized based on formative research studies, pretesting of materials, feedback during early implementation, and field observations. BF IPC implementation was through existing well‐established government and NGO maternal and child health services, in alignment with pre‐existing national policies. Service delivery differed across countries from almost all community‐based in Bangladesh to mostly facility‐based in Vietnam; the details are published elsewhere (A&T 2012, 2013; BRAC 2013; Kim et al. 2015; Nguyen et al. 2014, 2021; Sanghvi et al. 2016). The total duration of program activities, including planning, research, strategy development, materials development and production, training, delivery, and monitoring of interventions was 4.7 years in Bangladesh, 4 years in Ethiopia, and 4.1 years in Vietnam. The duration of exposure to BF IPC was 46 months in Bangladesh, 30 months in Ethiopia, and 36 months in Vietnam.

During implementation, the program content and implementation strategies were intended to be adjusted if indicated by monitoring and supervision feedback. Most programs used data from a combination of self‐monitoring embedded in service registers and special rapid assessments conducted by external locally hired teams. In Bangladesh, rapid assessments of coverage and BF practices and in Ethiopia, a process evaluation survey was conducted. Vietnam developed a more sophisticated routine monitoring system for government health centers to track new and returning clients that received franchised counseling services. Country differences in program content, structure, and management arrangements reflected differences in program needs, existing service delivery capacity, barriers to early initiation and exclusive BF, and health service utilization practices. Bangladesh implemented the BF IPC services in almost half the subdistricts nationally and Ethiopia implemented BF IPC in four of the most populous regions. Vietnam implemented an innovative franchise model in 15 of 63 provinces selected from all geographic regions and developed a community support group model for hard‐to‐reach underserved ethnic areas.

## 2 Methods

We used a top‐down, aggregate costing method based on accounting records maintained for program expenditures in each country to calculate the incremental financial costs based on expenditures of BF IPC. Bangladesh, Ethiopia, and Vietnam were selected for this analysis based on supportive national policies, the large scale of efforts to strengthen programs, and impacts of interventions used for strengthening existing services on BF practices as documented by rigorous external evaluations (Kim et al. 2016; Menon et al. 2016). Despite pre‐existing guidelines, country BF indicators had not demonstrated improvements before the initiatives included here. The expenditures represent a payer perspective and include BF IPC expenditures incurred over and above existing BF services including personnel already working in program areas. The methodology is an accounting of incremental financial expenditures rather than an economic analysis of costs (Belli 2001). The aim was to provide country planners and managers with a realistic calculation of what to budget for strengthening large‐scale programs to provide BF counseling to mothers. The methodology involved separating BF IPC activities and expenditures incurred from broader infant and young child feeding (IYCF) programs that involved mass media/public education and complementary feeding as well as BF. Almost all mass media/public education activities were distinct from IPC activities and implemented by different implementing agencies; however, a proportion of expenditures for formative studies and management that were jointly done were allocated proportionately in consultation with country implementers. To separate BF IPC from complementary feeding IPC we took into consideration the emphasis and focus of each country program based on a content review of materials, discussions with implementing staff, and documentation by external evaluation partners. In Bangladesh, both BF and complementary feeding received equal emphasis. In Ethiopia, IPC focused mainly on complementary feeding (EBF was already high), and in Vietnam, BF received substantially more inputs (dietary diversity was already high). To separate operational subcomponent expenditures, the accounting system for expenditures maintained the data by vendor or implementer who were contracted for executing specific operational components. We aggregated the expenditure data and compared the total expenditures, average expenditures per year, expenditure shares of operational components, and expenditure per participant reached in each country. The databases for program expenditures were similar across the countries and this permitted us to make cross‐country comparisons of total and program component expenditures.

### 2.1 Estimating Incremental Financial Costs or Expenditures

Countries were assigned a unique code by the global accounting team at the start of the programs in 2009–2010, and each procurement transaction for goods and services was also coded to a program component that was consistent across countries. We extracted data on payments made to agencies contracted for implementing BF IPC activities. All expenditures incurred were included, such as for formative studies and assessments used to develop strategies and plan implementation activities; designing, testing, printing, and disseminating tools and materials; building the capacity of frontline workers to deliver skilled BF counseling; conducting group activities and community events; administrative and management tasks; and supervision and monitoring. We entered information extracted from invoices paid to vendors and implementers into our accounting databases. All expenditures were converted by accounting managers to USD using the prevailing exchange rates. Conversion into 2022 USD was done using the Consumer Price Index ratio of the year of expenditure to the Consumer Price Index (CPI) in 2022, and CPI data was obtained from the World Bank World Development Indicators available online at https://data.worldbank.org/products/wdi.

The total expenditures included all BF IPC program activities that were implemented through ANC, delivery and postpartum services, and infant/child health services at facilities, through community outreach, and at community and household levels. The activities aimed to support pregnant and nursing women with newborns and infants through the efforts of health workers and community and family members. We aggregated the cost components to calculate the total incremental costs of each country program. Program reports provided the total program durations. The total expenditures incurred over the life of each country program were divided by the number of project years to calculate the average annual expenditures per year.

### 2.2 Calculation of the Number of Participants

#### 2.2.1 Number of Women and Infants

The number of BF IPC participants consists of the number of PW in the last two trimesters and infants below 12 months and their mothers who were reached by the interventions as measured through program coverage results and population data. External evaluations were conducted in a subset of program areas; interventions in evaluation areas are termed as ‘high intensity’ and in non‐evaluation intervention areas are termed ‘low intensity.’ Bangladesh and Ethiopia strengthened existing maternal and child health services and Vietnam added a community support group program in marginalized areas in addition to strengthening existing health services. The number of PW, mothers and infants residing in program areas was obtained from program monitoring data, 2012–2014 national and regional population data, program records and World Bank population databases, accessed at https://data.worldbank.org/indicator/SP.POP.TOTL. Program monitoring data and impact evaluation results provided percent coverage for each country (Kim et al. 2015, 2016; Menon et al. 2016).

#### 2.2.2 Number of Influential Persons

In addition to PW and mothers, we included five categories of influential persons that the programs needed to engage for facilitating scaled‐up counseling services for PW and mothers and for the protection/adoption of recommended breastfeeding practices by mothers. In all country programs, the number of health personnel trained in BF counseling and equipped with counseling tools was included; family members were included assuming one additional adult per PW and mother. Based on the knowledge of community dynamics in each country, we calculated community opinion leaders in program areas at one per 500 mothers in Bangladesh, one per 1000 households (approximating a cluster of communities or a Kebele) in Ethiopia, and three per community support group in Vietnam. Religious leaders in IPC areas were actively engaged by the Bangladesh and Ethiopia programs and calculated at 1 per 500 mothers in Bangladesh and Ethiopia. We calculated peers at one per four mothers in Bangladesh and Ethiopia, and one per two mothers in Vietnam. The sum of these five groups yields the total number of influential persons reached for supporting BF IPC services and protection/adoption of BF practices.

### 2.3 Calculation of Unit or Per Participant Expenditures

We used the average annual total expenditure per year and participants reached per year to calculate the unit expenditures in terms of the expenditure per PW, mother and infant reached, and the expenditure per PW, mother, infant and influential person reached. We divided the total annual expenditure by the number of participants reached each year, using the numbers of PW, mothers and infants reached; and the numbers of PW, mothers, infants and influential persons reached.

### 2.4 Sensitivity Analysis

We identified the calculation of participants reached in low‐intensity IPC areas where evaluation surveys were not conducted as an area of uncertainty. To reduce the possibility of overestimating the number of PW, women, and infants and the number of PW, mothers, infants, and influential persons we discounted the total participant estimates by 15% and compared the base scenario with the unit expenditures using discounted numbers for participants reached.

### 2.5 Ethics Statement

The collection of cost data did not involve human participants. The coverage data were previously published, and ethical approval was obtained from IFPRI's institutional review board in accordance with the guidelines laid down in the Declaration of Helsinki.

## 3 Results

### 3.1 Total and Annual Expenditures

**Figure 1:**
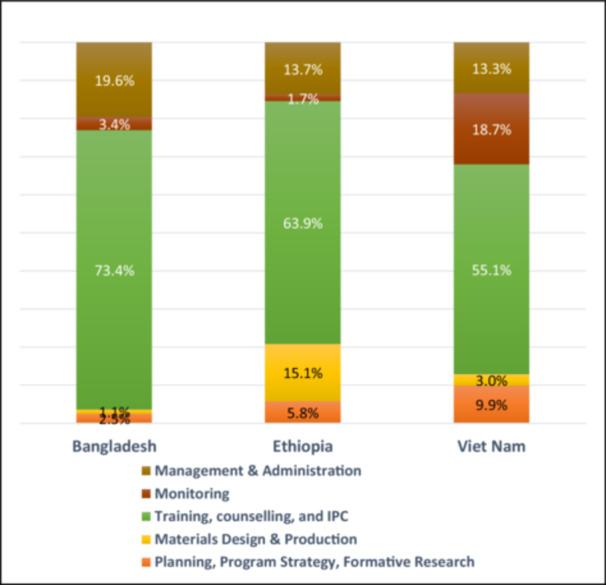
Proportional cost shares of program components by country (%).

The incremental financial costs documented using expenditure data in this paper reflect the budgetary expenditures needed to bring existing BF efforts to levels where measurable increases in BF IPC coverage and impacts on practices can be achieved. The total expenditures for scaled‐up BF IPC programs were USD 3,230,121 over 4.7 years in Bangladesh, USD 1,873,401 over 4 years in Ethiopia, and USD 3,183,868 over 4.1 years in Vietnam (Table 1). The programs involved differing levels of planning, strategy development and formative research; materials development and production; and strengthening of health systems and community platforms to deliver individual and group IPC. The largest proportion of expenditures were incurred for training and delivering BF IPC in all countries ranging from 55.1% in Vietnam to 63.9% in Ethiopia and 73.4% in Bangladesh (Figure 1). As a guide for budget planning, the average expenditures by program components in the three country programs were USD 168,091 for planning, strategy development and formative studies; USD 137,153 for materials development and production; USD 1.774 million for training, service delivery and supervision of BF IPC provision; and USD 437,025 for management and administration. Expenditures on monitoring activities were quite different from over USD 594,000 in Vietnam (likely due to the special requirements of a unique innovation being tested) to USD 31,259 in Ethiopia (likely to be inadequate) to USD 111,298 in Bangladesh.

**Table 1:** Total and annual breastfeeding IPC costs by program component and country (2022 USD).

Program component	Bangladesh	Ethiopia	Vietnam
Total program duration (period of exposure)	4.7 years (46 months)	4.0 years (30 months)	4.1 years (36 months)
Planning, strategy, formative research	$80,533	$107,834	$315,907
Materials design and production	$33,992	$282,097	$95,370
Counseling, training, community mobilization	$2,372,469	$1,196,415	$1,754,709
Program monitoring	$111,298	$31,259	$594,434
Management and administration^a^	$631,830	$255,796	$423,449
Total costs	$3,230,121	$1,873,401	$3,183,868
Annual program costs	$687,260	$468,350	$776,553

^a^ Some management & administration costs were loaded on other cost elements and are reflected in the rows above.

### 3.2 Participants Reached

#### 3.2.1 PW, Infants Below 1 Year and Their Mothers

The number of women and infants was 3.03 times higher in Bangladesh (1,241,306) per year as compared with Vietnam (409,115), and 1.76 times higher than Ethiopia (720,086) (Table 2). It reflects the high coverage of existing community‐based health workers and volunteers in Bangladesh who counseled on BF, and health extension workers in Ethiopia based in health posts close to communities. Bangladesh and Ethiopia delivered BF IPC through home visits while Vietnam required PW and mothers of infants to visit health facilities.

**Table 2:** Participants reached annually with breastfeeding IPC.

Variable	Bangladesh^1^	Ethiopia^2^	Vietnam‐franchise^1^	Vietnam‐CSG^3^
Pregnant women, mothers and infants reached				
High‐intensity IPC areas (evaluation)				
Numbers of PW, infants and mothers residing in high‐intensity areas	519,701	669,882	758,572	77,586
Percentage reached by IPC	85%	45%	48%	58%
Number reached by IPC	441,746	301,447	364,115	45,000
Low‐intensity IPC areas				
Numbers of PW, infants and mothers residing in high‐intensity areas residing in low‐intensity areas	1,881,318	1,550,513	na	na
Percentage reached by IPC	43%	27%	na	na
Number reached by IPC	799,560	418,639	na	na
All IPC areas				
Mothers & children residing in areas receiving any IPC	2,401,019	2,220,395	836,158	
Mothers & children reached by IPC	1,241,306	720,086	409,115	
Influential persons reached				
(i) Health personnel (program records)	75,000	21,000	16,500	
(ii) Family Members, assuming 1 per PW and mother‐child dyad reached by IPC (1 dyad = 1/2 of mothers and infants < 6 months)	689,608	400,047	227,286	
(iii) Community Leaders, assuming 1/500 PW and dyads reached by IPC in Bangladesh, 1/1000 in Ethiopia, 3 per Support Group in Vietnam	1379	400	801	
(iv) Religious Leaders, assuming 1 per 500 PW and dyads reached by IPC intervention in Bangladesh and Ethiopia	1379	800	—	
(v) Peers, assuming 1 per 4 PW and dyads in Bangladesh and Ethiopia; 1 per 2 PW and dyads in Vietnam reached by any intervention	172,402	100,012	113,643	
Total number of influential persons reached, sum of (i) through (v) above	939,768	522,259	358,230	
Total participants reached				
Pregnant women, Infants, mothers, and influentials	2,181,074	1,242,345	767,345	

Abbreviations: CSG = community support group, IPC = interpersonal communication, PW = pregnant women.

1 Coverage data for Bangladesh and Vietnam (franchises) was measured through RCTs; in Vietnam's CSGs and Ethiopia, coverage was calculated from the plausibility evaluation, monitoring, and process evaluation studies. Influential persons reached are based on formative studies and program descriptions of interventions implemented to reach specific categories of influential persons. REFERENCES 1. (Menon et al. 2016) 2. (Kim et al. 2016) 3. (Nguyen et al. 2021).

#### 3.2.2 Influential Persons

The programs incurred expenditures to identify key categories of influential persons, developed materials for each, and engaged various networks and institutions to reach them. The number of influential persons ranged from approximately 400,000 to 900,000 each year (Table 2).

### 3.3 Expenditure Per Participant Reached

The expenditures per participant were calculated by dividing the annual expenditures by the number of participants reached each year, resulting in a range of USD 0.55 to USD 1.90 per PW, mother and infant reached annually; and USD 0.32 to USD 1.01 per influential person and PW, mother and infant reached annually (Table 3). These modest per participant expenditures apply to large scale programs and reflect substantial economies of scale. They are also only the incremental costs or expenditures for strengthening existing services for BF IPC. Higher expenditures per participant in Vietnam reflect the challenges in reaching large numbers of PW, mother and infants through services provided almost entirely through government health facilities, as compared with community‐based service delivery including private providers. As this analysis was from a provider perspective, it does not consider transportation, time and opportunity costs of PW and mothers to obtain BF IPC services. The expenditure per PW, mother and infant reached with BF IPC differed by 3–3.5 times across countries; the expenditure per all participants reached including influential persons and PW, mothers and infants differed by 2.7–2.9 times.

**Table 3:** Unit costs of breastfeeding IPC interventions (2022, $ USD).

Variable	Bangladesh	Ethiopia	Vietnam
Program costs (USD)			
Total program costs, Table 2	$3,230,121	$1,873,401	$3,183,868
Annual program costs, Table 2	$687,260	$468,350	$776,553
Number of participants reached in program areas annually			
PW, infants < 12 months and mothers reached by IPC, Table 3	1,241,306	720,086	409,115
Total number of participants (PW, infants, mothers, influential persons), Table 4	2,181,074	1,242,345	767,345
Unit costs per participant reached (USD annual)			
Cost per PW, infant, and mother reached	$0.55	$0.65	$1.90
Cost per all participants reached, including influential persons	$0.32	$0.38	$1.01

Abbreviations: IPC = interpersonal communication, PW = pregnant woman.

### 3.4 Sensitivity Analysis

Accounting for uncertainties in country program coverage by reducing the population reached by 15% resulted in increased annual costs per PW, mother, and infant from USD 0.55 to USD 0.65 in Bangladesh, from USD 0.65 to USD 0.77 in Ethiopia, and from USD 1.90 to USD 2.23 in Vietnam (Table 4). Similarly, the annual expenditure per key participant increased due to a lower denominator across the countries, from USD 0.32 to USD 0.37 in Bangladesh, USD 0.38 to USD 0.44 in Ethiopia, and USD 1.01 to USD 1.19 in Vietnam. The implications for budgeting are that countries need to invest USD 0.55 to USD 2.25 per PW, mother, and infant to deliver BF IPC in their catchment area. Based on the differences in program models and sites where BF IPC was delivered, the engagement of community outreach processes that already reach this age group with high coverage on a large scale can reduce the cost per PW, mother and infant reached substantially.

**Table 4:** Sensitivity analysis: 15% Reduction in participants reached by breastfeeding IPC interventions.

Participants reached	Bangladesh	Ethiopia	Vietnam
Base scenario, Tables 3 and 4			
PW, infants, and mothers	1,241,306	720,086	409,115
PW, infants, mothers and influential persons	2,181,074	1,242,345	767,345
Alternate scenario: 15% lower reach			
PW, infants, and mothers	1,055,110	612,073	347,748
PW, infants, mothers and influentials	1,853,913	1,055,993	652,243
Program costs (USD)			
Annual program costs, Table 2	$687,260	$468,350	$776,553
Unit costs of participants reached (USD)			
Base scenario, Table 5			
Cost per mother/child reached	$0.55	$0.65	$1.90
Cost per mother/child/influential reached	$0.32	$0.38	$1.01
Alternate scenario: 15% lower reach			
Cost per mother/child reached	$0.65	$0.77	$2.23
Cost per mother/child/influential reached	$0.37	$0.44	$1.19

## 4 Discussion

We documented the total incremental financial costs based on expenditures incurred, number of beneficiaries, and expenditure per participant of three large‐scale BF IPC intervention programs using retrospective accounting of expenditures and participant coverage measured through RCT evaluations. The incremental financial cost or expenditures on BF IPC ranged from USD 0.55 in Bangladesh to USD 0.65 in Ethiopia to USD 1.90 per PW, infant, and mother reached, differing by types of service delivery and levels of BF IPC utilization by participants. To our knowledge, this is the first detailed comparative analysis of BF IPC delivered at scale with details of program models, expenditures, cost structures, and reach/coverage in diverse LMICs ranging from Bangladesh and Ethiopia to Vietnam (Carroll et al. 2020). The results are consistent with the program models where Vietnam leveraged existing government health facilities to position intensified BF IPC services, while Bangladesh and Ethiopia invested more resources in outreach to deliver BF IPC in communities and homes. Bangladesh utilized one volunteer cadre of community workers plus two salaried cadres of trained IPC providers while in Ethiopia, salaried government health extension workers were trained, equipped, and supervised to deliver BF IPC. In Vietnam, a unique innovative model was designed for embedding franchises into government primary health care centers and required extensive formative work; it also involved elaborate monitoring to document the franchise model's multiple operational characteristics, which is reflected in a higher proportion of total expenditures incurred for strategy design and monitoring as compared with Bangladesh and Ethiopia where the focus was on strengthening ongoing BF IPC services.

The program contexts and time taken for planning, strategy development, testing and implementation differed by country. In Bangladesh, an extensive system of home visits and community engagement formats developed by a national nongovernmental organization (BRAC) already existed and it cost a total of USD 3,230,121 over 4.7 years covering all phases of program development and implementation. In Ethiopia implementation was conducted through government health posts, community group meetings led by government workers, and home visits by health extension workers across diverse regions and was implemented over 4 years at a cost of USD 1,873,401. In Vietnam, the program represented an innovative franchise model application for infant and young child nutrition services and was implemented through government health centers at district, provincial, and commune levels and through community support groups. This required extensive planning, design, testing, development, and monitoring; the Vietnam program was conducted over 4.1 years at a cost of USD 3,183,868.

In addition to the women and infants serviced directly, the study quantified the number of influential persons engaged to improve effective BF counseling services at scale and illustrates ‘the causal chain of support’ for PW and mothers who ultimately choose whether and how to put their newborn to their breast at birth and continue BF (Lutter and Morrow 2013; Pérez‐Escamilla et al. 2023). Our calculations show that across countries, 800,000 PW, mothers, and infants on average, and 650,000 influential persons were reached annually. The impact on BF practices of the counseling component alone could not be measured, since additional interventions in particular mass media, public education, and policy advocacy are likely to have contributed to the improvements in BF indicators (Kim et al. 2016; Menon et al. 2016). The importance of BF IPC alone or in combination with other interventions has been documented, however, and global recommendations call for universal coverage of all PW and mothers of infants with BF IPC (United Nations Children's Fund (UNICEF), World Health Organization 2021).

The costing based on expenditures not only provides a benchmark for budgeting but can also inform the design of more efficient program components and decisions on how and where to invest to strengthen service delivery, conduct formative research and strategy development, design and produce tools and materials used for implementation, and monitor programming (Hutubessy et al. 2002). By comparing country programs, this study illustrates both the efficiencies and diverse programmatic needs of country programs. For example, the choice of venues for delivering BF counseling needs to align with health services utilization patterns. The sophistication of training methods and counseling tools reflect the educational levels of providers and mothers and have cost implications.

Among program components, the largest proportion of expenditures in all countries were incurred for counseling mothers at 64% of the total on average, including the training of multiple cadres of IPC providers, the establishment of counseling sites at health facilities, and group meetings and events at a community level. Administrative and management expenditures were 15% of the total expenditures on average; planning/strategy development and formative research, materials design and production, and monitoring, each accounted for between 6% and 8% of total expenditures. Some program component expenditures differed more across countries than other components. For example, the dollar amounts for service delivery (counseling mothers, training, and community and facility activities) differed the least with a twofold difference between the highest and lowest cost country programs. Administration and management expenditures differed 2.5‐fold, planning/strategy development and formative research fourfold; and materials design and production eightfold. Bangladesh incurred the smallest expenditures in dollar amounts for materials design and production largely due to stakeholder requests for only a single pocket size ‘reminder card’ for counseling providers with a list of bullet points instead of more elaborate counseling cards and flip charts that were already available and found too bulky; furthermore, no materials were distributed to mothers and families in Bangladesh unlike in other countries. As for the expenditure on monitoring, the additional monitoring requirement in Vietnam for a new innovative franchise model was likely one of the drivers of the 19‐fold higher monitoring expenditure incurred as compared to the lowest amount spent on monitoring in Ethiopia, although Ethiopia may not have invested sufficiently in monitoring.

Our findings contribute to supporting the financing and country‐wide implementation of one of the most cost‐effective child survival and newborn health and nutrition interventions globally, in addition to providing insights about program components to facilitate more efficient designs of future BF IPC programs (Carroll et al. 2020; Victora et al. 2016). The country differences in program component expenditures suggest the need for contextualizing interventions and considering ranges of expenditures while budgeting for BF IPC (Carroll et al. 2018; Gustafsson‐Wright and Boggild‐Jones 2018).

Cost studies of BF programs available in the literature seldom use comparable methods and rarely provide details of program interventions (Carroll et al. 2020). Several studies have used modeling and hypothetical inputs and provided broad financial approximations for planning at a global level but these have limited relevance for planning country‐specific program component budgets, or they focus on one component such as training (Arslanian et al. 2022), or on a single location (Menon et al. 2016; Silva et al. 2021), or do not clearly indicate budgetary needs for countries to strengthen existing BF IPC. For example, the scaling up of a BF counseling package was theoretically projected for the KwaZulu‐Natal province of South Africa (Desmond et al. 2008). In Uganda, a community‐based peer counseling program's annual intervention costs of a more intensive and less intensive model ranged from USD 139 to USD 74 per mother, respectively. In Vietnam, the community support group model in this study was independently costed out and found that the financial cost from the providers' perspective for supporting the meetings, compensating village collaborators, and providing supportive supervision through staff in commune health stations was USD 5 per PW or mother, and increased to USD 15 when training, supervision, and additional administrative costs at central and provincial levels were added (Nguyen et al. 2021). The smaller scale and higher intensity of community support groups likely accounted for the higher unit costs as compared with the overall Vietnam program documented here.

In addition to BF IPC, the three study programs utilized national mass media and regional public education channels to reinforce BF IPC messages among influential persons that were needed to shift norms, and remind and motivate mothers/caregivers and frontline workers about what desirable BF practices are, why they are critically important, and how to achieve them within the prevailing birthing practices, maternal time constraints, and traditional misperceptions such as the need for water in warm weather before 6 months of age. External rigorous evaluations found that combined intervention exposures led to larger improvements in BF as compared with BF IPC or BF media exposure individually (Kim et al. 2020; Menon et al. 2016). The unit costs of the mass media components of Bangladesh and Vietnam programs were calculated at USD 0.13 per woman and infant in Bangladesh and USD 0.38 in Vietnam (Sanghvi et al. 2022). The lower unit costs as compared with unit costs of BF IPC were likely due to high media coverage and large numbers reached, for example, an estimated 5.57 million women and infants in Bangladesh and 3.19 million in Vietnam were covered annually. Evaluations showed a dose–response relationship with each additional type of exposure reported by mothers increasing the likelihood of improving BF practices. This indicates that national BF program budgets should ideally include multiple interventions for changing BF indicators on a large scale to reach Sustainable Development Goals.

Our three‐country study provides incremental financial costs per beneficiary based on expenditures and provides a rough benchmark for LMICs interested in investments to strengthen their existing BF support programs. In addition to aiding budget planning, our expenditure analysis may be used to prepare investment cases and estimating ‘global price tags’ (Vassall et al. 2018). For example, according to a recent model, the gap in current BF practices in Bangladesh results in 3150 child deaths and 500 maternal deaths each year, and losses due to additional health care costs equal USD 3.4 million in addition to cognitive losses and future earnings (Walters et al. 2019). Scaling up BF globally could prevent illnesses and an estimated 820,000 deaths of children under five globally, 20,000 breast cancer deaths among mothers each year, and reduce hypertension and diabetes in women (Rollins et al. 2016; Victora et al. 2016).

We are unable to attribute the improved BF practices documented through external evaluations solely to IPC because public education and mass media were used to drive IPC reach by promoting service utilization through mass media and IPC providers were more motivated and knowledgeable when exposed to mass media and this translated into better IPC results (Menon et al. 2016; Nguyen et al. 2019). Limitations of the study include not considering societal or participant costs; however, the aim of the paper is to elucidate program budgetary expenditures from the providers' perspective for strengthening existing services. Our archived accounting records on expenditures did not provide input level costs, but gross amounts spent on intervention components; for this reason, we included detailed descriptions of activities in this paper and recommend ingredients‐based costing to be conducted prospectively in the future.

Finally, Victora's observation two decades ago that cost‐effective public health interventions are not reaching developing country populations who need them is still valid for BF IPC (Victora et al. 2004). Additional investments are needed and as shown in our three‐country expenditure analysis, building on known, effective health services with proven interventions can help to reach universal coverage. The availability of sufficient funds is essential, based on accurate estimates of resource requirements and resources are urgently needed for strategic planning based on formative work, and for intervention delivery by strengthening health system components that are required for BF IPC including counseling at scale. The rationale for universalizing BF counseling and strategies and tools for delivering results at scale are already available.

## Author Contributions

Conceptualization: Tina G. Sanghvi and Rick Homan. Methodology, Rick Homan, Tina G. Sanghvi, Marina Nersesyan. Validation: Patricia Preware, TW, Zeba Mahmud, Tuan Nguyen, and Roger Mathisen. Formal analysis: Marina Nersesyan, Rick Homan and Tina G. Sanghvi. Resources: Tina G. Sanghvi, Marina Nersesyan, and Rick Homan. Data curation: Patricia Preware, Zeba Mahmud, Tuan Nguyen and Roger Mathisen. Writing – original draft preparation: Tina G. Sanghvi and Rick Homan. Writing – review and editing: Tuan Nguyen, Patricia Preware, Marina Nersesyan, TW, Zeba Mahmud, Edward A. Frongillo, and Roger Mathisen. Supervision: Tina G. Sanghvi and Rick Homan. Project administration: Tina G. Sanghvi, Rick Homan and Marina Nersesyan. Funding acquisition: Marina Nersesyan. All authors have read and agreed to the submitted manuscript. All authors read and approved the final paper.

## Conflicts of Interest

The authors declare no conflicts of interest.

## Supporting information

rev Supplementary table Oct 13.

## Data Availability

The data that support the findings of this study are openly available in International Food Policy Research (IFPRI) dataverse at https://dataverse.harvard.edu/dataverse/IFPRI. Expenditure data are in the manuscript tables. Datasets for coverage data are anonymized and available on A&T's repository on Harvard Dataverse.
