# Supplementary material for: Expenditures on Strengthening Large Scale Breastfeeding Counseling Programs in Bangladesh, Ethiopia, and Vietnam
Source: Matern Child Nutr. 2025 Apr 4;21(3):e70031. doi: 10.1111/mcn.70031 (PMC12150148; doi:10.1111/mcn.70031)
Supplement: Supplementary file 1 — rev Supplementary table Oct 13. [file MCN-21-e70031-s001.docx]

***Supplementary***

**Table: Content of Breastfeeding Interpersonal Communication Programs Across Countries**

| **Characteristics** | **Bangladesh**  **2009-2014** | **Ethiopia**  **2009-2014** | **Vietnam**  **2009-2014** |
| --- | --- | --- | --- |
| **Interpersonal Communication (IPC) Interventions** | | | |
| **Content focus** | Early initiation < 1 hour, no pre-lacteals, use of colostrum, EBF for 6 months, continued BF as part of complementary feeding after 6 months. Teaching of frequency, appropriate attachment and position of breastfeeding, manual expression, and not giving water or other liquids to maintain breastmilk supply. Also, self-assessment of breastmilk adequacy through counting number of urinations. | Early initiation < 1 hour, no pre-lacteals, use of colostrum, EBF for 6 months, continued breastfeeding after 6 months. Pre-emptive problem-solving: maintain supply of breastmilk such as by frequent breastfeeds; also, build-up the mother’s confidence of her ability to provide enough breastmilk for her child. | Early initiation < 1 hour, no pre-lacteals, use of colostrum, EBF for 6 months, continued breastfeeding as part of complementary feeding after 6 months. Special focus on delaying the introduction of complementary foods to extend EBF for 6 months, citing global recommendations, using images of celebrity mothers, and teaching manual expression of breastmilk if mother is separated. |
| **Protocols** | Monthly home visits by community volunteers (SS) assigned 250–300 households; health workers (SK, 1 per 20 SS) enroll PW, emphasize EIBF and EBF during ANC and PNC, counselling on appropriate position and attachment, orient family members at the first visit, address difficult cases. Nutrition workers (PK) supports SS (1:10), records visits, group education by SK for PW and new mothers. ^[1,2]^ | Counseling at health posts and home visits by HEW and health volunteers Women’s/ Health Development Armies (WDAs/HDAs); ‘Smart and Strong’ families was a key concept promoted; HEWs responsible for multiple programs; group discussions held at village gatherings led by HEWs, engagement of religious leaders and women’s associations. ^[3,4]^ | Counseling at franchised health centers and community groups (CSG) in remote areas; 8 contacts: 3 during pregnancy to prepare for EIBF and EBF, 1 at/after delivery, 4 to maintain BF. Both in group and individual sessions at the franchise; leaflets, mother-and-child booklets, and promotional items (face cloths, raincoats). CSGs for peer dialog and problem solving by trained health workers ^[5]^ |
| **Service delivery sites and visits** | Monthly home visits for pregnant women and through 2 years; group counseling at community level for pregnant women, after delivery and postnatally ^[6]^. | Health posts and home visits for pregnant women and mothers through 2 years; ‘community conversations’ (groups); visits by religious and women leaders ^[4]^. | Facility-based counseling in franchised health centers at community, district, provincial levels; CSG at village level in hard-to-reach areas/ethnic groups ^[7,8]^. |
| **Counseling providers and facilitators** | 3 NGO (BRAC) cadres: health volunteers (SS) and nutrition workers (PK), MNCH providers (SK); tasks include family engagement; 75,000 BRAC and other NGO workers and TBAs trained ^[9,10]^. | Government-salaried health extension workers (HEWs) and community health volunteers Women’s Development Army (WDA) or Health Dev. Army (HDA); religious and women leaders 21,000 FLWs trained ^[10,11]^. | Nurses, midwives, nutritionists, trained to deliver IYCF counseling, and village health workers (VHWs) motivated mothers to attend franchises. Facilitators for support groups. 16,500 FLWs and VHWs, managers trained ^[10,12]^. |
| **Training** | BRAC FLWs and supervisors trained using an illustrated manual plus 15-min. video to standardize content across 16 regional training sites; 2 days supervised field practice. Quarterly refresher training for HW, monthly for volunteers, and orientation and refresher training for TBAs. Problem-solving skills based on monitoring and supervision feedback ^[1]^. | Several sources of training for MOH staff including IFHP and REST provided to HEWs and volunteers through cascade approach, IYCF added to information and materials into the maternal and child health module of national Integrated Training. Problem-solving skills and using IEC materials developed based on monitoring and supervision feedback ^[13,14]^. | Manual and handbook used to provide essential skills for counseling and communication to health workers at health facilities. Manual and handbook also for VHWs to generate demand for franchise services. Orientation sessions for trainers, government district and PHC staff. How to use counseling materials and problem-solving skills reinforced based on monitoring and supervision feedback ^[12]^. |
| **IPC tools** | Illustrated job aids used as reminders for counseling providers (individual and group education) on age-specific messages for counseling mothers; included common BF problems and solutions. Posters at health facilities used during scale up ^[15]^. | Child Nutrition Card with brief messages and images on EIBF and EBF used at health facilities, community meetings and home visits, also intended for in-home display. Challenges in distribution at community level ^[16-18]^. | Counseling cards and videos (e.g., breastmilk production, position, attachment) in counseling sessions. Posters on franchise walls to reinforce key messages, booklets and leaflets distributed to clients. Website developed for problem-solving and to engage fathers ^[12]^. |
| **Supervision** | Community health volunteers supervised monthly by health and nutrition workers; subdistrict and district managers used data on coverage at monthly feedback meetings; checklist for counseling observations used by supervisors ^[1]^. | 52.5% of HEWs in Tigray and 33.7% in SNNPR reported supervisory visits, volunteers 41.8% in Tigray and 21.8% in SNNPR in the past one month; low quality of support and problem-solving reportedly for HEWs ^[16]^ | Supervision protocol and checklists used in social franchises. Counseling providers reported satisfaction with supervisors’ flexibility, support, and response to their concerns. Supervision improved the FLW performance through better motivation. ^[19]^ |
| **Community, family engagement** | Volunteers and HWs promoted BF practices to family members in home visits; additional community meetings held for fathers and community and religious leaders and other elites. | Group discussions or ‘community conversations’ facilitated by the WDAs/HDAs and HEWs for mothers and community members to generate support for EIBF, EBF. | VHWs tasked with identifying and encouraging mothers to seek IPC at facilities. Community support groups expanded the reach of counseling; fathers peer support generated by social media. |
|  |  |  |  |
| **Behavior- change strategies for more channels and more frequent contacts with mothers and influential persons, and to address barriers and enablers** | Training and frequent follow up with 3 cadres of workers providing frequent face to face contacts from pregnancy to 6 months. Influential members of the family and community engaged through networks. Village birth attendants and doctors reached through mass media; provided problem-solving tactics on EBF through media for health workforce ^[6,21]^. | Promoting more frequent health post visits motivated by HEW and volunteers; increased home visits and engaging influential family and community members (evidence of dose response with impact on EIBF). Tracking through monitoring system. Combining health worker with community-based activities for enhancing impact; use of local media ^[13,16,18]^. | Preparation during ANC for EIBF, staff attending newborn care and PNC trained to facilitate skills-building among pregnant women and mothers, and motivation for 6 months of EBF. Husbands engaged in supporting mothers, media and internet promotion, celebrity role models, mothers taught to express breastmilk if separated due to work; legislators, MOH, WHO endorsement ^[19,22,23]^. |
|  | | | |
|  |  |  |  |
|  |  |  |  |
|  |  |  |  |
|  |  |  |  |

BRAC= Bangladesh national NGO, EBF= exclusive breastfeeding, EIBF=early initiation of breastfeeding within the first hour after delivery, CM=community mobilization FLW=frontline worker, HDA=Health Development Army, HEW=health extension worker, HW=health worker, IFHP= Integrated Family Health Project, IPC=interpersonal communication, MOH=Ministry of Health, NGO=non-governmental organization, , SNNPR= Southern Nations, Nationalities and Peoples’ Region, VHW=village health worker, WDA=Women’s Development Army.

REFERENCES FOR SUPPLEMENTARY TABLE

[1] BRAC. *Implementation Manual: Community-based IYCF Program, BRAC Bangladesh* 2013.

[2] Menon P, Nguyen PH, Saha KK, et al. Impacts on Breastfeeding Practices of At-Scale Strategies That Combine Intensive Interpersonal Counseling, Mass Media, and Community Mobilization: Results of Cluster-Randomized Program Evaluations in Bangladesh and Viet Nam. *PLoS Med*. Oct 2016;13(10):e1002159. doi:10.1371/journal.pmed.1002159

[3] Kim SS, Rawat R, Mwangi EM, et al. Exposure to Large-Scale Social and Behavior Change Communication Interventions Is Associated with Improvements in Infant and Young Child Feeding Practices in Ethiopia. *PLoS One*. 2016;11(10):e0164800. doi:10.1371/journal.pone.0164800

[4] A&T. *Moving From Nutrition Crisis to Nutrition Security: Alive & Thrive’s Approach and Results in Ethiopia*. 2015.

[5] Nguyen PH, Mai LT, Rawat R, Menon P. *Viet Nam Impact Evaluation 2014 Final Report*. 2015.

[6] AliveThrive. *Alive & Thrive Less Guess: Getting Strategic with Interpersonal Communication*. 2015.

[7] Nguyen PH, Kim SS, Keithly SC, et al. Incorporating elements of social franchising in government health services improves the quality of infant and young child feeding counselling services at commune health centres in Vietnam. *Health Policy Plan*. Dec 2014;29(8):1008-20. doi:10.1093/heapol/czt083

[8] Nguyen TT, Hajeebhoy N, Li J, Do CT, Mathisen R, Frongillo EA. Community support model on breastfeeding and complementary feeding practices in remote areas in Vietnam: implementation, cost, and effectiveness. *Int J Equity Health*. May 17 2021;20(1):121. doi:10.1186/s12939-021-01451-0

[9] BRAC. *Scaling Up and Sustaining Support for Improved Infant and Young Child Feeding: BRAC’s Experience through the Alive & Thrive Initiative in Bangladesh*. 2014.

[10] A&T. *Milestone Tables for the period of December 1, 2008 to August 31, 2017 (Phases 1-2)*. 2017.

[11] A&T. *Improving child feeding practices to prevent malnutrition: Alive & Thrive’s approach and results in Ethiopia*. 2014.

[12] A&T. *Toolkit for infant and young child feeding counseling services: A social franchise model, Vietnam*. 2015.

[13] A&T. *Timed and age appropriate messaging approach on infant and young child feeding in Ethiopia*. 2017.

[14] A&T. *Multi-media and Training Catalogue*. 2017.

[15] A&T. *Print & Audio Visual Materials for Promoting Infant and Young Child Feeding, Bangladesh*. 2014. *Catalogue*.

[16] Kim SS, Ali D, Kennedy A, et al. Assessing implementation fidelity of a community-based infant and young child feeding intervention in Ethiopia identifies delivery challenges that limit reach to communities: a mixed-method process evaluation study. *BMC Public Health*. 2015/04/01 2015;15(1):316. doi:10.1186/s12889-015-1650-4

[17] A&T. *Timed and Age-Appropriate IYCF Messaging for Health Development Army Team leaders (HDATLs) and Health Extension Workers (HEWs) in Ethiopia* 2012.

[18] A&T. *Promoting young child feeding with the Ethiopian Orthodox Church*. 2017.

[19] Nguyen PH, Kim SS, Tran LM, Menon P, Frongillo EA. Intervention Design Elements Are Associated with Frontline Health Workers’ Performance to Deliver Infant and Young Child Nutrition Services in Bangladesh and Vietnam. *Current Developments in Nutrition*. 2019/08/01/ 2019;3(8):3008001. doi:<https://doi.org/10.1093/cdn/nzz070>

[20] Kim SS, Tesfaye R, Kennedy A. *Ethiopia Impact Evaluation Summary Report* 2014.

[21] Kim SS, Nguyen PH, Tran LM, Alayon S, Menon P, Frongillo EA. Different Combinations of Behavior Change Interventions and Frequencies of Interpersonal Contacts Are Associated with Infant and Young Child Feeding Practices in Bangladesh, Ethiopia, and Vietnam. *Curr Dev Nutr*. Feb 2020;4(2):nzz140. doi:10.1093/cdn/nzz140

[22] Nguyen PH, Kim SS, Nguyen TT, et al. Exposure to mass media and interpersonal counseling has additive effects on exclusive breastfeeding and its psychosocial determinants among Vietnamese mothers. *Matern Child Nutr*. Oct 2016;12(4):713-25. doi:10.1111/mcn.12330

[23] Nguyen PH, Kim SS, Nguyen TT, et al. Supply- and Demand-Side Factors Influencing Utilization of Infant and Young Child Feeding Counselling Services in Viet Nam. *PLoS One*. 2016;11(3):e0151358. doi:10.1371/journal.pone.0151358

[24] Frongillo EA. Designing and implementing at-scale programs to improve complementary feeding. *Nutr Rev*. Dec 1 2020;78(Suppl 2):62-70. doi:10.1093/nutrit/nuz043
